# A Climatic Stability Approach to Prioritizing Global Conservation Investments

**DOI:** 10.1371/journal.pone.0015103

**Published:** 2010-11-30

**Authors:** Takuya Iwamura, Kerrie A. Wilson, Oscar Venter, Hugh P. Possingham

**Affiliations:** Ecology Centre, The University of Queensland, Brisbane, Queensland, Australia; Smithsonian's National Zoological Park, United States of America

## Abstract

Climate change is impacting species and ecosystems globally. Many existing templates to identify the most important areas to conserve terrestrial biodiversity at the global scale neglect the future impacts of climate change. Unstable climatic conditions are predicted to undermine conservation investments in the future. This paper presents an approach to developing a resource allocation algorithm for conservation investment that incorporates the ecological stability of ecoregions under climate change. We discover that allocating funds in this way changes the optimal schedule of global investments both spatially and temporally. This allocation reduces the biodiversity loss of terrestrial endemic species from protected areas due to climate change by 22% for the period of 2002–2052, when compared to allocations that do not consider climate change. To maximize the resilience of global biodiversity to climate change we recommend that funding be increased in ecoregions located in the tropics and/or mid-elevation habitats, where climatic conditions are predicted to remain relatively stable. Accounting for the ecological stability of ecoregions provides a realistic approach to incorporating climate change into global conservation planning, with potential to save more species from extinction in the long term.

## Introduction

Habitat loss has historically posed the greatest threat to biodiversity [1], yet climate change is now impacting species and ecosystems globally [2]. If today's rate of warming continues, we are likely to witness a serious climate-induced decline in the world's biodiversity during this century [3], [4]. Protected areas have played a key role in safeguarding biodiversity from habitat loss [5]. There is accumulating concern that the future effectiveness of protected areas will be undermined by climate change for the conservation of biodiversity [6], [7], [8], [9] (but see [10]).

Over the past few decades, biodiversity conservation has become increasingly global in scale [11]. The number of projects led by international initiatives such as United Nation's Environmental Program has increased dramatically [12]. Major conservation NGOs (e.g. The Nature Conservancy (TNC), World Wildlife Fund (WWF), Conservation International (CI), and the International Union of Conservation Nature (IUCN)) have also developed international portfolios of investment [13]. Global financing frameworks, such as Global Environment Facility, have also established large budgets to conserve biodiversity at the global scale [14].

Global resource allocation schemes that prioritize international conservation investments [15], [16] guide the spending of at least US $1.5 billion per annum [17]. International NGOs have agreed upon the boundaries of unique ecosystems (termed ‘ecoregions’), which are increasingly used as the spatial unit considered for prioritization [18]. Ecoregions are defined as “a relatively large unit of land or water containing a characteristic set of natural communities that share a large majority of their species, dynamics, and environmental conditions” [18]. Global prioritization schemes typically use insights from regional and local conservation practices, such as irreplaceability and vulnerability [5], [19]. Increasingly, these schemes account for the costs of conservation [20], [21] and most include a measure of threat, typically the rate of land conversion [15], [22]. Despite research indicating that climate change is a major emerging threat to biodiversity [3], and one that could undermine conservation investments if not accounted for [8], no existing prioritization scheme at the global scale has considered the possible impacts of climate change on biodiversity [15]. Given the extent to which climate change is predicted to affect biodiversity and compromise conservation investments, it is imperative that the impacts of climate change are considered explicitly when allocating funds for conservation at a global scale.

Methods for incorporating climate change into conservation planning have been developed for regional scale planning [6], [23], [24]. The majority of these approaches are based on the prediction of species range shifts and use correlative species distribution models [25] which predict species ranges using occurrence or abundance data and environmental variables. However, overcoming differences among model predictions [26] and discrepancies between fundamental and realized niches [27] usually requires detailed data on species migration rates, interspecies interactions, and rates of adaptation [28]. The utility of conservation prioritization based on future species ranges remains constrained by our ability to compile and analyse these data for thousands of species over large spatial scales. It is therefore worthwhile to seek an alternative approach to maximizing the chance of conserving existing ecosystems in the face of rapid climate change.

This paper presents a novel approach to allocating conservation resources under a changing climate at the global scale. Climate change is predicted to affect the stability of climate [7] and undermine the effectiveness of conservation investments to protect species [29]. Our approach focuses on the climatic stability of ecological regions for terrestrial endemic vertebrates and identifies priority areas predicted to be robust to the impacts of climate change. It incorporates the impacts of climate change into conservation planning without depending on predicting species' future ranges. Our method can be applied to any geographical scale, but it is particularly suitable to the global scale where the different predictions of species ranges less likely agree with each other [30]. In this paper, we demonstrate the implementation of our approach by allocating conservation resources among global ecoregions. We assess how investment scheduling to maximize species persistence should shift in space and time to incorporate the effects of climate change.

## Materials and Methods

We used a dynamic resource allocation algorithm [31], [32] to prioritize conservation investments across the world's ecoregions to ensure persistence of biodiversity in the future. The problem the algorithm solves is to minimize species loss by reducing the expected negative impact of climate change and land conversion on vertebrate species, constrained by a fixed budget and accounting for the area already protected [33] or developed [34]. This paper demonstrates how to allocate conservation funding of 20 years (2002–2022) to achieve highest biodiversity persistence in 2052.

The current and future climate profiles of the world's terrestrial ecoregions [35] were evaluated to develop a measure of their ecological stability under climate change. The proportion of each ecoregion potentially affected by future climate change was then used to infer the potential impact of climate change on 6,777 terrestrial vertebrate endemic species [36]. A lack of data at the ecoregion scale precluded our incorporating a wider suite of taxa. The proportion of area potentially affected by future climate change in each ecoregion was used to develop a measure of an ecoregion's ecological stability under climate change. We assumed that conservation investments are used to establish new protected areas [37], the cost of which was estimated using foregone agricultural rents [38]. Land conversion rates were calculated from the historical record of croplands and farmlands expansion between 2000 and 2005 based on HYDE 3.0 dataset [39], [40].

We allocated investments under two resource allocation scenarios: a climate-adapted allocation and a climate-neutral allocation. In the climate-adapted allocation, the algorithm discounts the expected benefit of investing in an ecoregion by the probability that the invested area is affected by future climate change, thereby giving increased value to ecoregions that are predicted to remain ecologically stable in the face of climate change. This probability is set to zero across all the ecoregions in the climate-neutral allocation. We then measured how the allocation of funds shifted in space and time by incorporating the predicted negative effects of a changing climate.

### Climate dataset

Observed spatial databases of global temperature, precipitation, cloud cover, vapour pressure, and diurnal temperature range were downloaded from the “CRU TS 2.10” dataset of the Tyndall Institute, UK [41]. For each climate variable, we calculated yearly averages from the monthly data from1997 to 2002. Maximum and minimum values for temperature or precipitation were not used, as they did not affect the results of Principle Component Analysis (PCA) due to their strong correlations with the averages. Estimated spatial databases of the same climate variables from 2047 to 2052 were from the “TYN SC 2.03” dataset [41]. We constructed the estimated climatic variables in future climate based on four major General Circulation Models (GCMs; HadCM3, CSIRO mk 2, DOE PCM, and CGCM2) for each of the four IPCC greenhouse gas emission scenarios (A1fi, A2, B1 and B2) [42].

The original resolution of both the observed and estimated dataset is 0.5 Arc degrees. We could include only 680 ecoregions for prioritization at this resolution, because some ecoregions are smaller than a minimum of three data points (7,500 km^2^) which are required to create a convex hull polygon (see below). To produce more data points, and thus include 791 ecoregions (minimum approx. 300 km^2^), we interpolated the data at resolution of 0.1 Arc degrees with the Inverse Distance Weighted (IDW) interpolation method. This was performed in ArcGIS9.3 with the power set to the conventional value of 2.0 and 24 data points were used to estimate the value. Downscaling climate data has been undertaken previously [43], however this was for a smaller number of parameters and greenhouse gas emission scenarios. The sensitivity of the allocation to interpolation method was investigated by also using Spline interpolation. For the Spline interpolation, we used Spline tool of ArcGIS 9.3 with the “Regularized” option and 0.1 weight value (see Results section).

### Climatic stability

**Figure 1:**
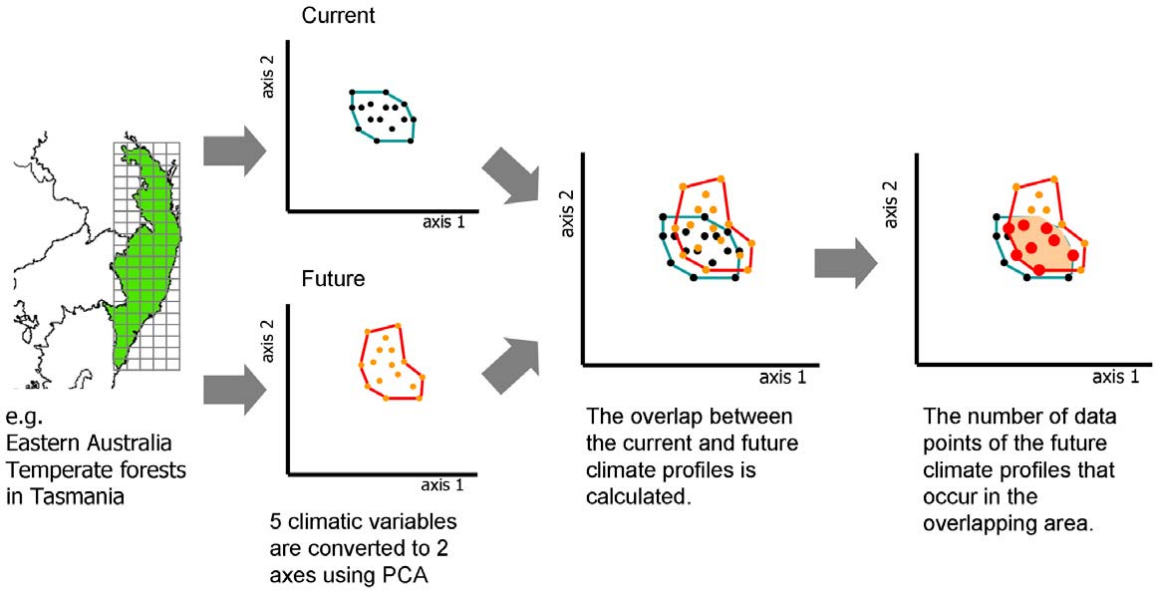
Methods to calculate climatic stability. An ecoregion's climatic stability is defined as the proportion of the parcels in an ecoregion which are predicted to remain climatically stable. The example is shown for the Eastern Australia Temperate Forests ecoregion.

An ecoregion's climatic stability *Si* is defined as the proportion of the parcels in ecoregion *i* which are predicted to remain climatically stable. A parcel is considered climatically stable if its predicted (2047–2052) climatic conditions remain within the current (1997–2002) climate profile of the ecoregion (Figure 1).

The current climate profile of each ecoregion was determined based on spatial datasets for five climate variables: annual mean temperature, precipitation, cloud cover, vapour pressure, and diurnal temperature range [41]. Using the first two axes from principal component analysis, we developed the current climate profile of each ecoregion as a two-dimensional convex hull polygon (Figure 1). We excluded climate data from land classified as already developed (GLC 2000 dataset [34]). Observed data was projected on to PCA variable space with the princomp function of R (http://www.r-project.org/).

Estimated climate variables were projected onto the same variable space with the covariance matrix obtained from the observed dataset. The value for the *n* ^th^ axis of PCA variable space for the cell *j* of ecoregion *i*, 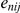, was calculated based on the standardized climate variables and the eigenvectors of covariance matrix gain through observed climate profiles for all the climate types:
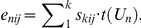
where 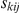 was standardized to the range of (0,1) from the climate variable *k* (of the 5 variables describe above) of the cell *j* within ecoregion *i*. The function 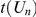 is the transposed eigenvector for *n* ^th^ axis of PCA variable space, calculated from the observed dataset.

The two major axes (n = 1 and 2) of the PCA score for the observed and the estimated climatic variables were used to calculate the 2-dimensional convex hull polygon. These two scores explain more than 95% of variance of all the climate variables (the cumulative proportion of variance is 81.7% with the first axis and 95.6% with the first and second axes).

The same covariate matrix from the current climate profile was applied to construct the future climate profiles of each ecoregion based on the average of the estimated future climate variables from the four GCMs [41] under each of the four IPCC greenhouse gas emission scenarios (A1fi, A2, B2 and B1) [44].

### Dynamic resource allocation algorithm

Each ecoregion was divided into parcels of 0.1km^2^. A parcel can be in one of the three states: 1) available 2) reserved or 3) degraded. If a parcel is available, it can be selected by the algorithm to be reserved. If it is not selected for reservation, it can be affected by either climate change or land conversion. If the parcel is reserved, it is no longer vulnerable to land conversion but remains susceptible to climate change. Parcels affected by either climate change or land conversion are then considered degraded and remain in that state. The allocation runs for 25 time steps, with each time step representing one year. The total budget is set to the 2.5% of the total cost of the world's available land surface. All parcels are assumed to hold the same cost, contain the same ecoregion specific values of climatic stability, biodiversity benefit, and land conversion rate.

The algorithm selects parcels to minimize the probability of species loss until the annual budget is exhausted. Specifically, the algorithm allocates funds to maximize 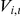,which represents the increased biodiversity value if the parcel within ecoregion *i* is invested in at time *t*, while the total investment should be smaller than the yearly budget, where
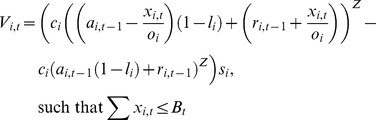
where 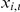 is the amount of investment in ecoregion *i* at time step *t*, 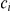 is a constant within the species area relationship, calculated using the total number of species and the size of ecoregion *i* over *z*. *z* gives the shape of the species area relationship [45]. We set *z* to be 0.2 across all the ecoregions. The sensitivity of our results to this parameter was evaluated. 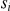 is the probability of climatic stability, which represents the probability that a parcel of ecoregion *i* remains suitable for native species. 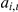 is the total available area in ecoregion *i* at time *t*. 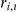
is the total area reserved in ecoregion *i* at time *t*. 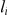 is the land conversion rate for ecoregion *i*. 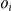 is the opportunity cost of protecting land in ecoregion *i*. 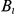 is the size of available budget at year *t*.

We evaluated the impact of climate change on the location and timing of investments. The timing of investments was recorded when the accumulated investment in an ecoregion reaches 0.5 percent of total budget. We then considered an ecoregion to more urgently require investment if this threshold is met earlier in the climate-adapted allocation than in the climate-neutral allocation and less urgent if it occurs later. The conservation performance of an allocation schedule was examined by measuring species loss within protected areas due to climate change and species loss in the overall landscape, which are calculated based on 100 simulations where land conversion, climate change and reserve establishment occur annually.

## Results

**Figure 2:**
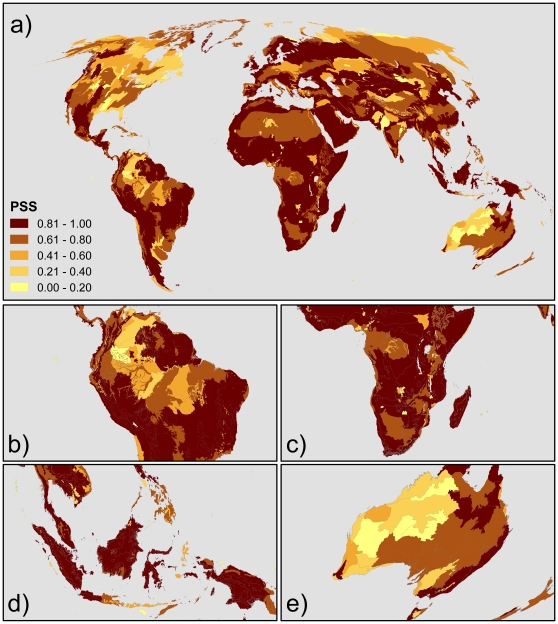
The climatic stability of ecoregions under the A1fi IPCC emission scenario. The result is based on the average climatic stability from 4 different GCMs. The darker colors represent more stable climates. The climatic stability (a) at the global scale. (b–e) at regional scales; (b) of the Amazon Basin. Western part of Amazon shows higher climatic stability than flood plain. (c) of Madagascar and southern Africa. Montenous area in the East Africa have higher climatic stability. (d) of South east Asian island and New Guinea. The mid elevation areas in this region show very high climatic stability. (e) of eastern Australia. Sub tropical forest has much higher climatic stability than inland desert area.

Figure 2 shows the relative differences in the climatic stabilities among the 791 ecoregions due to differences in ecoregion's climatic stability. It is observed that there is substantial spatial variation in the predicted climatic stability of ecoregions. The ecoregions of high stability largely overlap with the areas known for their high biodiversity. These areas include the Andes, tropical savannas in Africa, Madagascar, islands in South East Asia (Borneo, Java, and Sulawesi), New Guinea, the western coast of the Indian subcontinent, and the subtropical forests of the east coast of Australia, where the climatic stabilities are more than 80%. The areas with less stability are found in north and middle-western North America, the west of Amazon basin, Siberia, Himalayas, and the tropical savannah and desert regions of Australia.

**Figure 3:**
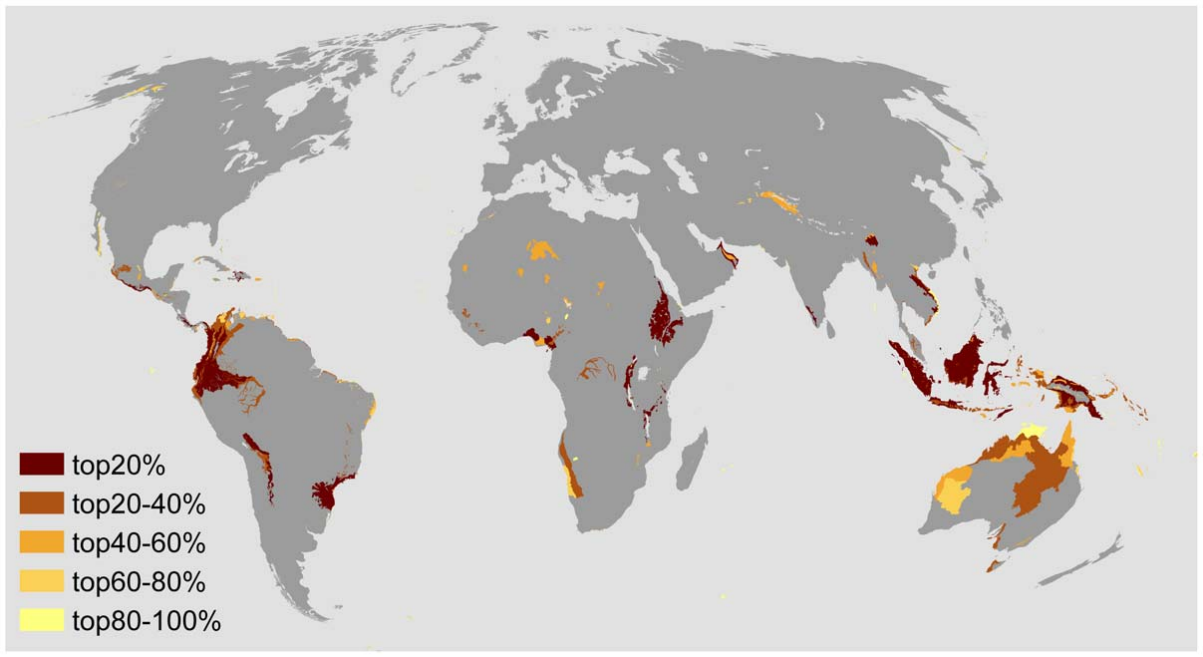
The pattern of accumulated investments. The result is to minimize species loss from land conversion and climate change under the 20 years allocation with the budget level of 2.5% of the total cost of the available land surface. The categories represent 5 quantiles of the investment amount (top 20%, 20–40%, 40–60%, 60%–80% and 80%∼), where the ecoregions within the top 20% group receive most investment in this particular allocation. The A1fi IPCC emission scenario was applied.

**Figure 4:**
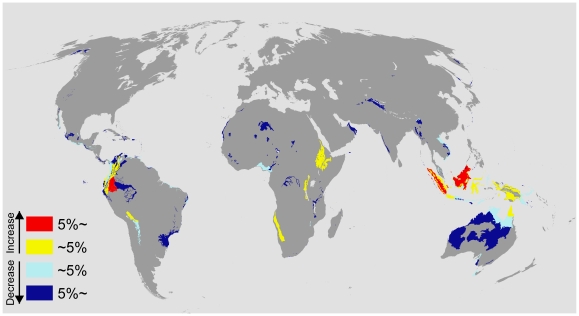
Differences in the total investment under the “climate-adapted” and “climate-neutral” allocations. Given is the % of the change in their investment within an ecoregion. “Warm” colors (yellow and red) represent the ecoregions that receive increased investment under climate-allocation. “Cold” colors (pale blue and dark blue) represent the ecoregions with decreased investment. The result for the A1fi IPCC emission scenario is displayed. The budget is 2.5% of the total cost of the available land surface.

Figure 3 shows the optimal allocation of conservation investments to reduce species loss from land conversion under a changing climate. Incorporating the predicted impacts of climate change results in a substantial spatial shift in conservation investments (Figure 4). Specifically, 9 percent of the total budget was shifted to different areas between the climate-adapted allocation and the climate-neutral allocation. The quantity of funding shifted was robust to the IPCC greenhouse gas emission scenarios, with a maximum deviation of only 1.3% from the average. This is because the variation of climatic stability amongst the scenarios is smaller than the variance among ecoregions within each scenario. We only describe the results from the A1fi scenario as it represents the worst case prediction, assuming rapid economic and population growth based on fossil fuel as the main energy resource.

**Figure 5:**
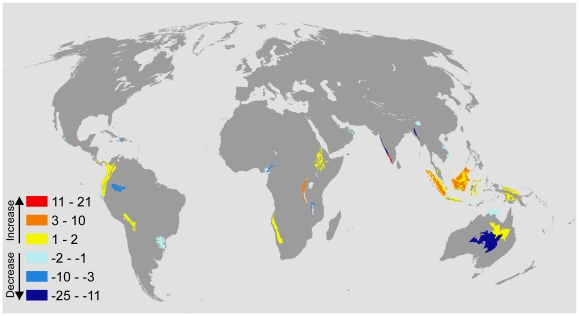
Differences in the timing of investment under the “climate-adapted” and “climate-neutral” scenarios. The categories indicate the relative investment urgency when we assume a changing climate. Dark red indicates that investment to the ecoregion is more urgent (and the number of years earlier is indicated). Dark blue indicates that investment is delayed. The results for the A1fi IPCC emission scenario is displayed. The budget is 2.5% of the total cost to conserve the available land surface.

The ecoregions which received the greatest increase in investment in the climate-adapted allocation were located on the islands of South East Asia (e.g. Borneo, Sulawesi, and New Guinea) and some of the moist montane forests in the Andes and highlands of Eastern Africa (Figure 4). In the same time, the central west coast of Africa, Himalaya, dry forests in Mexico, and northern Australia all received less funding when we accounted for climate change (Figure 4). We found that accounting for the climatic stability also shifted the timing of investments to ecoregions (Figure 5). Twenty ecoregions become more urgent under the climate-adapted allocation and 28 become less urgent. The changes in investment timing were spatially similar to the changes in investment size, except for the western tropical savannah in Australia, where the timing of investments is more urgent but the total amount of investment is reduced (Figures 3 and 4).

**Figure 6:**
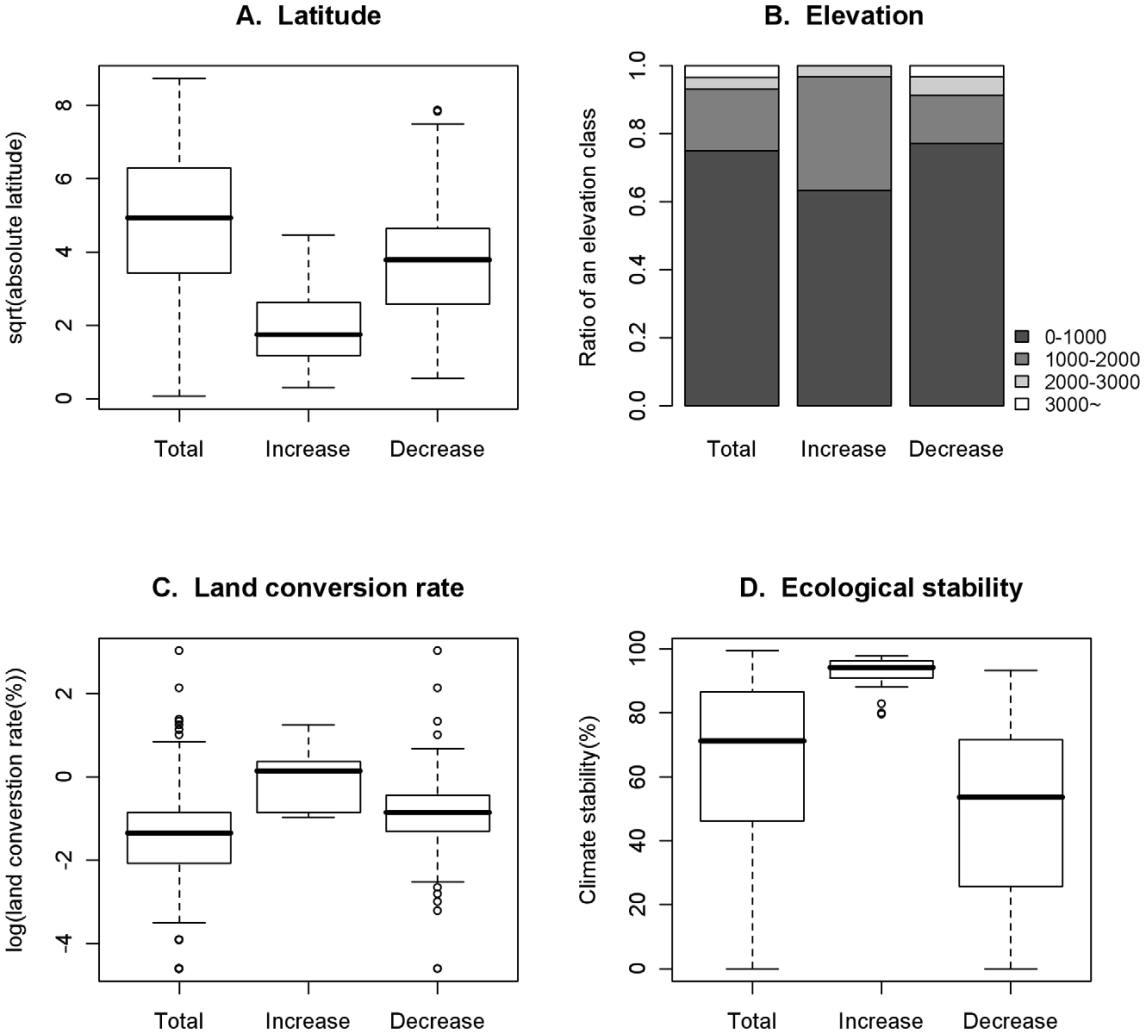
Trends in characteristics of ecoregions with increased investments. Total: all the ecoregions in the world. Increase: the ecoregions which increase investment under climate-adapted allocation. Decrease: the ecoregions for which the investment is reduced under the climate-adapted allocation. (a) latitude (square root transformed) (b) elevation classes (0–1000m, 1000–2000m, 2000–3000m and >3000m (c) land conversion rate (log transformed) and (d) Climatic stability.

When the climatic stability of existing ecoregions was taken into consideration, the ecoregions which received increased investment were more often located at lower latitude than those that received reduced funding (Figure 6a). The mean latitude of ecoregions with increased investments is 5°, whereas that of ecoregions with decreased investment is 18°. The ecoregions with increased investment were also generally located at mid elevations (between 1000–2000 m a.s.l.; Figure 6b). This trend is illustrated by the shifts in funding on the African continent. The investment on the west coastal lowland forests shifted to the montane forests in Cameroon, Ethiopia, and other parts of east Africa (Figures 3 and 4). The same trends in the shifts in latitude and elevation were also found in the analysis of investment timing.

Many of the ecoregions that received the greatest investment under the climate-adapted allocation were also identified in previous prioritization schemes (Figure 3). For instance, 86 percent of the ecoregions prioritized in the climate-adapted allocation were also identified as Biodiversity Hotspots by Conservation International and 87 percent are included in the Global 200 by WWF. Some important areas do emerge that are not currently considered as biodiversity hotspots. Most importantly among these are parts of New Guinea, Baja California and Australia's tropical savannah.

Incorporating the impacts of climate change reduces the chance of investing in areas that will become climatically unsuitable for indigenous species. Our analyses revealed that expected species loss from “protected” areas due to climate change was reduced by 22 percent on average in the climate-adapted allocation, compared with the climate-neutral allocation. A trade-off was observed, however, as some of the ecoregions highly threatened from land conversion become relatively less important under the climate-adapted allocation (Figure 6c). This was due to the reduced investment to the ecoregions with low climatic stability (Figure 6d). As a result, the species loss from land conversion increased 1 percent in the climate-adapted allocation. Overall, species loss from both protected and unprotected areas is reduced to 3 percent under climate-adapted allocation.

Sensitivity analyses were performed on model parameters for which information is most uncertain (Table S1). First, the uncertainties in climatic variables were examined. As described above, the results using four of the most commonly mentioned IPCC greenhouse gas emission scenarios did not vary much. Furthermore, the methods for downscaling GCMs were also examined. We applied two of the least similar methods for downscaling (see the Method section) and found that there was a 4 percent shift in investments between the two interpolations. Second, the sensitivity of investment pattern to the shape of the species area relationship was analyzed. The increase in species richness with increases in habitat area is known to follow a diminishing curve, but the shape of this curve can vary among regions [45], [46]. Varying the parameter *z* from 0.1 to 0.3 resulted in an average of a 0.8% change in investment in ecoregions.

## Discussion

This paper presents a novel approach to incorporating the impacts of climate change into large scale conservation prioritization. Currently-used prioritization schemes at a global scale generally measure the priority for investment as some function of the biological value and the vulnerability of the region [15]. While such analyses give a relative ranking of priorities, they do not provide guidance on the total proportion of funds that should be allocated to a region or the timing of the investments. Our approach considers the dynamics of land conversion [31], cost efficiency [20] and the probability of investment failure due to unstable climate, thus providing methods for spatially and temporally explicit resource allocation applicable to large scale prioritizations under climate change using currently available datasets.

Ecoregions that received increased investment under the climate-adapted allocation reflect their stable climatic conditions. In the Americas, for instance, investment shifted from arid ecoregions such as the dry forests in northern Mexico to wetter ecoregions such as Central Andean Wet Puna. This shift is supported by a general trend in which arid areas are considered to be particularly sensitive to the negative impacts of climate change [47]. Borneo, Sulawesi, and New Guinea all received increased funding in our climate-adapted allocation, which reflects the general view that the social factors (e.g. rapid land clearing) is the major threats in these areas while its climate remain relatively stable [48]. Our approach also suggests reducing investment in the Himalayas, an area which will experience severe negative impacts from climate change [42], [49].

Adapting our investments in protected areas to account for the future impacts of climate change ensures a more robust reserve network. In our scenario, we found that less area and fewer species will be lost from newly established protected areas by preferentially investing in climatically stable areas. Importantly, our modelling predicts that 22% more species remain within new protected areas in the climate-adapted allocation. Choosing to invest in areas with high climatic stability will ensure that our protected areas remain effective at conserving species in the face of climate change.

Mixed concordance was shown by comparing the observed trends of our investment allocation with that of the previous research based on predicted future species range. Both approach agreed on the shift in funding toward mid elevations [23]. This trend is supported by the fact that mid-elevations are where the species from higher altitudes and those from lower altitude overlap [50]. However, the priority on high latitudes of previous research is in contrast to our recommendations on emphasis in tropic regions. The investment trends in our approach reflects the comparatively stable climatic conditions in the tropics [51], [52] and the prediction that these areas will experience minimal climate change [53]. These areas, however, are unlikely to receive immigrating species from higher latitudes due to polewards nature of future species range shift [54]. Therefore prioritizations based on species migrations give reduced value to low latitude areas relative to higher latitudes, as the latter are typically predicted to receive immigrating species from the south [6], [9], [23].

These differences reflect a philosophical difference between the approaches. The method in this paper focuses on the stability of climate conditions, and values investments in areas which are predicted to remain suitable for indigenous species. It does not value lands which may receive immigrating species due to climate change. Therefore, the results shown in this paper are conservative with respect to the species gain associated with predicting where species will successfully immigrate and settle in the future. We note, however, that the techniques based on the prediction of species migration [6] and our approaches are complimentary, and together deliver a broader picture of the impacts from climate change. Our analyses at the global scale can function along with finer scale approaches where investment is further directed to smaller areas within an Ecoregion. We believe that existing regional scale techniques based on the prediction of future species ranges will be particularly effective in this context.

Our primary aim has been to propose a framework for global conservation prioritization that incorporates the impacts of climate change by focusing on climatic stability of ecological regions. The methods we used to calculate climatic stability could be improved by incorporating stochasticity, more climate variables and a wider suite of species. If other global threats, notably sea level rise, were accounted for, we expect that the funding to Ecoregions would shift, for instance due to reduced efficiency of protected areas in coastal areas. Our framework is open to incorporating more sophisticated methods to estimate the future impacts of climate change, and we recommend this as an area for future research. We also would like to emphasize that our approach can easily be modified to invest preferentially in areas where we predict high impacts from climate change, which may be desired if investments are in actions that are able to mitigate climate induced species loss, such as ecosystem engineering or translocation measures.

Our analysis places a focus on ecoregions where species loss from climate change is likely to be least while accounting for traditional issues such as cost, biodiversity and threat. This enables us to exercise the first global conservation scheduling that accounts for the risks associated with climate change. We find that by explicitly accounting for human-forced climate change we stand a greater chance of safeguarding biodiversity and ensuring the most efficient and effective use of the funds available for developing protected areas.

## Supporting Information

Table S1 **Sensitivity of key results to perturbation in model assumption and data.** Sensitivity was calculated as the percentage of the total investment shift in allocation. * In each randomization, the z value for each Ecoregion is drawn randomly from a uniform distribution with bounds of 01 and 0.5. 100 randomizations are performed.
(DOC)
