# Supplementary material for: A Climatic Stability Approach to Prioritizing Global Conservation Investments
Source: PLoS One. 2010 Nov 30;5(11):e15103. doi: 10.1371/journal.pone.0015103 (PMC2994894; doi:10.1371/journal.pone.0015103)
Supplement: Table S1 — Sensitivity of key results to perturbation in model assumption and data. Sensitivity was calculated as the percentage of the total investment shift in allocation. * In each randomization, the z value for each Ecoregion is drawn randomly from a uniform distribution with bounds of 01 and 0.5. 100 randomizations are performed. (DOC) [file pone.0015103.s001.doc]

**Supporting Information**

**Table S1. Sensitivity of key results to perturbation in model assumption and data.**

| **Model parameter** | **Perturbation** | **Sensitivity in results** |
| --- | --- | --- |
| Predictions of future climate variables (temperature, precipitation, cloud cover, diurnal temp difference, and vapor pressure) | IPCC greenhouse gas emission scenarios (A1fi, A2, B1, and B2) | ±1.3% |
| The method used to downscale climate data | Apply two distinctive interpolation: Inverse Distance Weighted interpolation and Spline interpolation | ±4% |
| The shape of Species-area relationship curve. z = 0.2 | Randomly perturb z between 0.1 to 0.3 * | ±0.8% |
